# Unraveling the Impacts of Germination on the Volatile and Fatty Acid Profile of Intermediate Wheatgrass (*Thinopyrum intermedium*) Seeds

**DOI:** 10.3390/molecules29174268

**Published:** 2024-09-09

**Authors:** Wellington S. Oliveira, Qianqian Chen, Dana Edleman, George A. Annor, Fernanda F. G. Dias

**Affiliations:** Department of Food Science and Nutrition, University of Minnesota, St. Paul, MN 55108, USA; dasilvaw@umn.edu (W.S.O.); chen8594@umn.edu (Q.C.); edlem002@umn.edu (D.E.); gannor@umn.edu (G.A.A.)

**Keywords:** intermediate wheatgrass, perennial crop, germination, volatiles, fatty acids

## Abstract

Intermediate wheatgrass (IWG) is a promising perennial grain explored for mainstream food applications. This study investigated the effects of different germination temperatures (10, 15, and 20 °C) and durations (2, 4, and 6 days) on IWG’s volatile and fatty acid (FA) profiles. A method using headspace solid-phase microextraction coupled with gas chromatography–mass spectrometry (HS-SPME-GC-MS) was optimized through response surface design to extract the volatile compounds, achieving ideal extraction conditions at 60 °C for 55 min. Multiple headspace extraction (MHE) was used for volatile compound quantification. Fifty-eight compounds were identified and quantified in IWG flour, mainly alcohols, aldehydes, hydrocarbons, terpenes, esters, organic acids, and ketones. The main FAs found were linoleic acid (C18:2), oleic acid (C18:1), palmitic acid (C16:0), and linolenic acid (C18:3). Principal component analysis showed a direct correlation between volatile oxidation products and FA composition. Germination at 15 °C for 6 days led to a reduced presence of aldehydes and alcohols such as nonanal and 1-pentanol. Therefore, optimized germination was successful in reducing the presence of potential off-odor compounds. This study provides valuable insights into the effects of germination on IWG flour, showing a way for its broader use in food applications.

## 1. Introduction

Grain crops can be divided into annual and perennial crops based on when their life cycles terminate. Annual grains, such as maize, rice, and wheat, have a short juvenile phase and rapid seed production that favors species survival [1]. On the other hand, perennial crops can be grown for several seasons and harvested annually, reducing the need for tillage, while their deep rooting habits help to increase soil carbon, water, and nutrients over time [1,2]. Given these benefits, perennials are increasingly considered more sustainable alternatives to annual crops, as they can contribute to more environmentally friendly farming practices.

Intermediate wheatgrass (IWG, *Thinopyrum intermedium*) is the first perennial grain crop developed and commercialized for human consumption. Developed at The Land Institute, the MN Clearwater is a food-grade IWG variety released in August 2019 and trademarked as Kernza^®^ [3]. In addition to the numerous advantages offered by perennial crops, IWG exemplifies cost efficiency in agricultural production. The reduced need for fertilizers and pesticides, coupled with the elimination of annual replanting, significantly lowers labor and maintenance expenses. This economical approach not only enhances the sustainability of farming practices but also contributes to greater financial viability for farmers [4].

Following its introduction, food products derived from IWG have increased in availability. Serving as a versatile substitute for wheat flour, IWG has been incorporated into a variety of products including bread, pancakes, beer, cereal, crackers, and cookies. Additionally, IWG can be utilized either as whole flour or in its germinated form, offering a range of culinary uses and nutritional benefits [5]. IWG was reported to be a great source of proteins (21%) and carbohydrates (73%), with a low fat (3%) and ash (2%) content [4].

Germination is the most basic process of growing plants from seeds. The process involves soaking dry grains in water, which encounters the physical conditions desirable for germination under humid conditions [6]. During germination, enzymatic activity increases, leading to the use of carbohydrates and lipids as energetic sources and impacting the formation of volatile compounds [7,8,9]. The process stimulates the seeds to re-establish various metabolic activities, leading to changes in biochemical, nutritional, and sensory characteristics [9].

Several factors impact germination, such as the variety of grain, temperature, humidity, presence of oxygen or air, light exposure, and pH [6]. Moreover, germination has been used to reduce undesirable flavors and enhance pleasant notes [8,10,11]. Only one study has reported the effect of different strategies such as germination and extrusion on the volatile profile of IWG [4]. In this study, germination was conducted under a single condition (16 °C for 4 days), and only qualitative data were reported for the volatile profile of IWG flour. While germination reduced the presence of off-notes such as 1-octen-3-ol, the lack of quantitative data prevents drawing definitive conclusions. A more comprehensive study examining the effects of various germination parameters on the volatile profile of IWG flour is essential. Quantitative data would facilitate better comparisons across different conditions and processes. Furthermore, no information is currently available on how germination influences precursors of off-notes, such as fatty acids (FAs). Thus, exploring the impact of germination on both the volatile and FA profiles of IWG flour warrants further investigation. Importantly, there are no studies dedicated to assessing the correlation between the quantitative profile of FA and volatile compounds during the germination of IWG at different days and temperatures.

Solid-phase microextraction (SPME) was introduced in the early 1990s and has become the most widely used technique for sampling volatile compounds in various applications, including food analysis, environmental studies, and metabolomics [12,13,14,15]. Several studies have already reported the use of SPME for profiling volatiles of germinated samples [16,17,18,19]. Most of them were performed using headspace (HS) sampling.

Quantifying volatiles using HS analysis is challenging due to the physicochemical properties of analytes, such as volatility, polarity, and partition coefficients. The physical state of the matrix (solid or liquid) and matrix effect further complicate the process [20].

Multiple headspace extractions (MHEs) arise as a solution for the aforementioned issue. This technique requires several extractions on the same sample, leading to exhaustive extraction [21]. The analyte concentration decays exponentially, and the total peak area can be obtained as the sum of each extraction [13,22]. Therefore, the analytes can be quantified using external standard calibration. MHE has not yet been applied for volatile profiling of germinated samples.

The overall objective of this study was to investigate, for the first time, the effects of different germination parameters on the FA and volatile profiles of IWG flour. Specifically, this work aimed to (i) investigate key germination parameters such as time (2, 4, and 6 days) and temperature (10, 15, and 20 °C); (ii) optimize SPME extraction conditions using a central composite rotatable design (CCRD) coupled with response surface methodology; (iii) assess the effects of germination on the IWG volatile profile; (iv) quantify aroma compounds using MHE, calibration curves, and gas chromatography coupled with mass spectrometry (GC-MS); and (v) assess the effects of germination on the FA profile of IWG flour by gas chromatography coupled with flame ionization detection (GC-FID). Correlations between the FA composition and the volatile profile were reported to better understand the changes occurring as a function of germination. This study provides valuable insights into the effects of germination on IWG flour, paving the way for its broader use in food applications.

## 2. Results and Discussion

**Figure 1:**
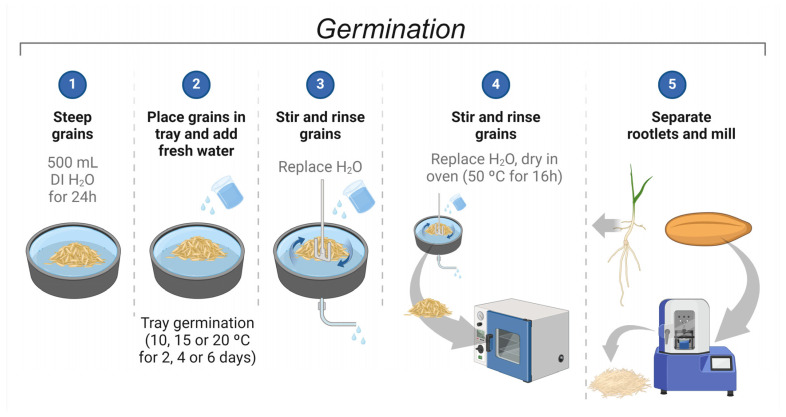
Diagram of the IWG germination process.

Germination has been used as a strategy to improve the overall quality of grains. During germination, plant hormones and enzymes are released, altering the nutritional and technological properties of the grain. Enzymes increase lipid oxidation, which can either increase or decrease off-flavors in germinated grains. To evaluate the effect of a controlled process on intermediate wheatgrass (IWG), germination was conducted using different temperatures and times (Figure 1) to assess their impact on the fatty acid and volatile composition of IWG.

### 2.1. Effects of Germination on the FA Profile of IWG

FAs play a crucial role in perennial grains by contributing to several key aspects of plant health, plant–plant, plant–microbe, and plant–environment interactions, nutrition aspects, and agricultural sustainability. They also play an important role in consumer acceptability as they serve as precursors to the formation of flavor compounds, such as aldehydes, that have a lower threshold [23].

The fatty acid profile of samples involves the extraction of lipids, derivatization of fatty acids, and chromatographic analysis of fatty acid methyl esters (FAMEs). For lipid extraction, solvent extraction methods such as the Folch or Bligh and Dyer methods are commonly used to isolate lipids from samples. These methods involve solvents such as chloroform and methanol to effectively extract fats and oils. To analyze FAs, they are often converted into fatty acid methyl esters (FAMEs) through transesterification or direct methylation. This makes the FA volatile and suitable for analysis using gas chromatography (GC). The primary technique for analyzing FAMEs is GC. FAs are separated and identified based on their retention times on a capillary column. The detection of the compounds is accomplished by a flame ionization detector (FID) (widely used in GC for detecting fatty acids) or mass spectrometry (MS) [24].

The overall FA profile of IWG samples exhibited a high concentration of linoleic acid (C18:2n6, ~62%), an essential polyunsaturated fatty acid (PUFA), followed by oleic acid (C18:1, ~17%) and palmitic acid (C16:0, ~12%) (Table S1—Supplementary Material). The total FA concentration varied from 16.41 to 21.78 mg/g of sample. This profile is similar to those found in wheat germ and sunflower oil, which present more than 60% of C18:2n6 in their composition. However, the content of C18:1 is higher in sunflower oil (28%) and lower in wheat germ (13%) when compared to IWG [25,26].

A significant reduction (from 13.76 mg/g on the control to approximately 10 mg/g after 6 days of germination) in the concentration of linoleic acid (C18:2n6) was observed in all temperatures evaluated (Table 1). Similarly, a reduction in the concentration of oleic acid (C18:1) and palmitic acid (C16:0) was observed within the germination process. However, the overall FA profile was not affected (Table S1). Germination time had a more significant effect on the FA content than the temperature (Table S1).

The germination process activates hydrolytic enzymes, which promote an increase in oligosaccharides, monosaccharides, oligopeptides, free amino acids, and free FAs by mobilizing them from the aleurone layer and scutellum to the starchy endosperm [9]. Therefore, a reduction in the FA content was expected during germination. Moreover, the depletion of FAs can be associated with their use as an energy source for protein synthesis in the growing vegetable [27].

Lipids are abundant in cereal tissues and can be used to provide energy during germination. The mobilization of triacylglycerol begins with germination, leading to the conversion of oil to sugars through the β-oxidation and glyoxylate cycles [28]. The process begins with the liberation of esterified FAs from triacylglycerols via lipase. Lipases are ubiquitous, being widely distributed in plants, animals (including insects), and microorganisms. They exhibit high specificity and selectivity for their substrates. In plants, the growing embryo has been reported to have the maximum lipase activity [29].

Loehr and co-workers [4] reported an increase in lipase activity in IWG. The authors found that within three months of storage, lipase activity significantly depleted triacylglycerols levels, leading to a peak in free FA content. These free FAs can serve as an energy source through β-oxidation and glyoxylate cycles during germination, ensuring the conversion of FAs into carbohydrates essential for seedling growth [30].

Lipoxygenase was also associated with the degradation of lipids during germination. Lipoxygenase catalyzes the oxygenation of linoleic acid (C18:2n-6) and other PUFAs containing the cis,cis-1,4-pentadiene moiety, forming monohydroperoxides with conjugated double bonds as primary products. It can act on free or esterified FAs [31].

Xu and co-workers [16] demonstrated that lipoxygenase activity increased after one day of germination in chickpea, lentil, and yellow peas, leading to the generation of volatile substances responsible for beany-related odors, such as (E)-2-octen-1-ol, hexanal, and 3-octen-2-one. The formation of these compounds showed a high correlation with the concentration of lipoxygenase and PUFAs.

Another factor that could be associated with the reduction in FA content is the temperature used for germination. Although temperatures of 10, 15, and 20 °C have not significantly impacted the concentration of FAs, the combination of enzyme activity and temperature should be considered. This combination can favor lipid oxidation and the formation of hydroperoxides [29]. Moreover, the IWG FA profile is mainly composed of linoleic acid (C18:2 n-6), which is more prone to oxidation than monounsaturated or saturated FAs. Bao et al. [32] pointed out that temperature plays a more significant role in the oxidation of C18:2 n-6 and the formation of volatiles than time. Furthermore, as observed during germination, most volatiles formed at lower temperatures have shorter carbon chains. In contrast, volatiles with longer carbon chains are more likely to be generated at higher temperatures. The short-chain compounds generated are potent odorants and can be associated with green, fatty, or vegetable-like flavors. Efforts should be made to avoid these off-notes in foods, as they are considered undesirable [33].

### 2.2. Effect of Germination on the Volatile Profile of IWG

#### 2.2.1. Fiber Selection

**Figure 2:**
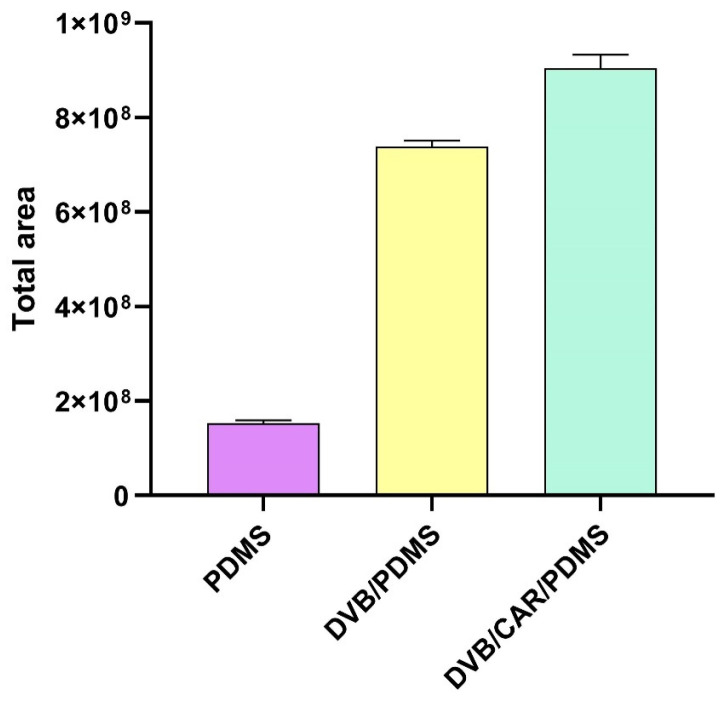
Extraction efficiency of the SPME fiber coatings tested. The results are expressed as the average of the triplicates for the total area of the chromatogram ± standard deviation for the volatiles from IWG by HS-SPME-GC/MS.

The choice of coating extraction phase is the first and most important step in the development of any SPME method since the fiber coating material determines the affinity between the analytes and the fiber [34]. The selection should be made considering the analytes, the sample, and the extraction mode [35]. For this study, three fibers (polydimethylsiloxane—PDMS, divinylbenzene/polydimethylsiloxane—DVB/PDMS, and divinylbenzene/carboxen/polydimethylsiloxane—DVB/CAR/PDMS) were evaluated regarding the suitability for the extraction of volatiles from IWG. Total area was used as the response (Figure 2).

The fiber composed of PDMS presented the lowest response for total area, while the fiber composed of DVB/CAR/PDMS showed the highest, with an area 5 times larger than that presented by the PDMS fiber. The DVB/PDMS fiber showed a total area 20% lower than the DVB/CAR/PDMS fiber.

Many factors contribute to the large variation in response to the different types of fibers. Firstly, the greater thickness of the triple fiber (80 µm) compared to the others (7 µm and 65 µm) achieves better extraction efficiency [36]. Secondly, the coatings have different affinities for volatile compounds and different extraction mechanisms, resulting in varying responses. PDMS is a liquid polymer that extracts volatile compounds based on the absorption of molecules inside the coating and has shown good efficiency in extracting nonpolar compounds. Conversely, in solid coatings such as CAR and DVB, extraction occurs only on the surface via an adsorption process through various interactions such as pi–pi bonding, hydrogen bonding, or van der Waals interactions [34]. When PDMS is combined with DVB, a bipolar fiber is achieved, allowing the extraction of both polar and nonpolar compounds [37]. Based on the best response obtained, the following optimization step was conducted using DVB/CAR/PDMS fiber.

#### 2.2.2. Optimization of Extraction Conditions

After fiber selection, a CCRD was performed to optimize the best extraction condition for the volatile compounds from IWG. The highest total area value (846,707,555) was achieved in experiment 6 (45 min of extraction time at 65 °C) (Table 2). Generally, an increase in the total area of the chromatograms is related to higher extraction temperatures. The increase in temperature shifts the equilibrium between the sample and HS [34], enhancing the concentration of volatiles in the HS.

Both time and temperature showed positive and significant impacts (α < 0.05) on the extraction of volatile compounds by SPME (Table 3). The analysis of variance indicated that only the linear factors had significant effects, without interaction between them. The regression model was evaluated using an F-calculated (Fcal) value, which was more than two times higher than the F-tabulated (Ftab) value, confirming the significance of the regression. Additionally, regarding the model’s lack of fit, the Fcal value was lower than the Ftab, confirming that the data fit a linear model. Moreover, the coefficient of determination was 0.92, indicating that the model can explain more than 92% of the data obtained in the experiment.

**Figure 3:**
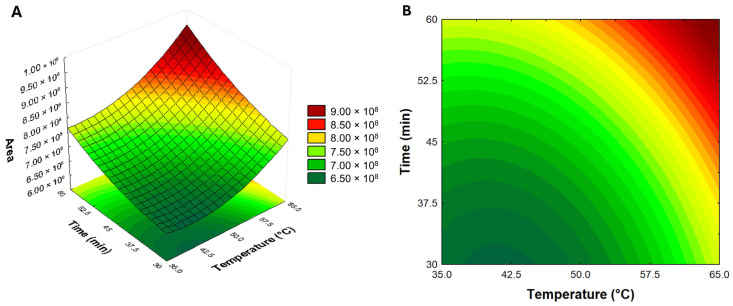
Response surface (**A**) and contour plots (**B**) of the impact of time and temperature on the total area of volatiles from IWG.

Considering these results, a response surface was used to visualize the best conditions to achieve maximum extraction of the volatile compounds (Figure 3). The surface response showed that the highest area values were achieved at temperatures above 60 °C and times longer than 55 min. Similar conditions were reported for the evaluation of roasted barley malts by HS-SPME-GC/MS [8]. The selected condition was used for the identification and quantification of volatiles from IWG by the MHE approach.

#### 2.2.3. MHE Optimization

The first step for optimization of the MHE was evaluating the amount of sample required. The area of volatile compounds should decrease by at least 5% between each extraction to achieve β values between 0.4 and 0.95 [38]. Values below 0.4 indicate that the analyte was fully extracted in the first extraction, making MHE unnecessary. Values above 0.95 suggest saturation of the HS due to high analyte concentrations, in which case the sample amount should be reduced to use MHE effectively.

**Figure 4:**
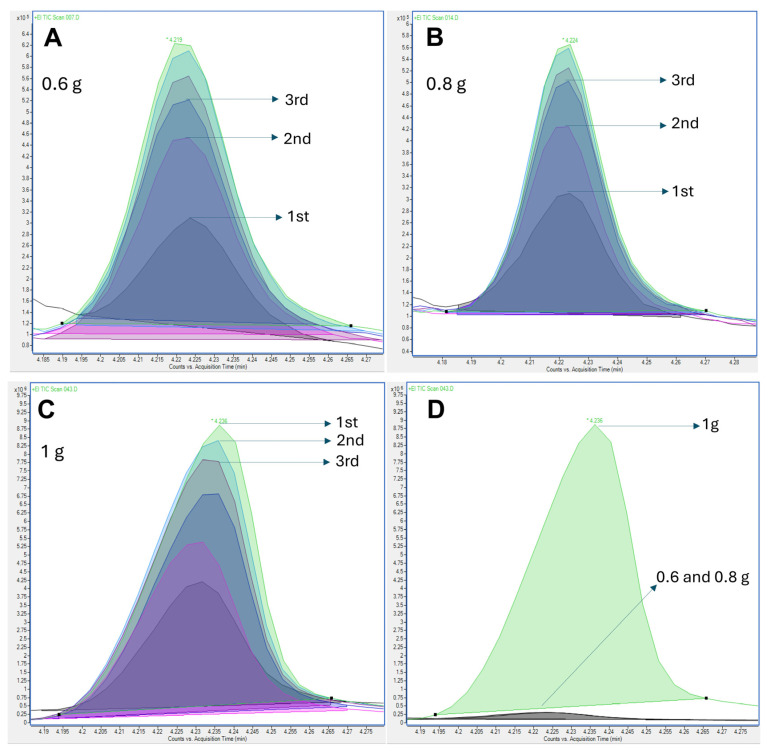
Decay after 6 extractions (**A**–**C**) and overlaid signals of the first extraction (**D**) for hexanal using 600, 800, and 1000 mg of sample.

Determining the amount of sample required to promote the decay for a multianalyte quantitation over a wide range of concentrations is a challenging task. For this evaluation, 600, 800, and 1000 mg masses were used and six extractions were performed. The sample size under study should be sufficient to release the minimum amount of analyte needed to match the method’s sensitivity and precision without saturating the HS [39]. The sample size of 600 and 800 mg were not sufficient to provide the exponential decay of the analytes (Figure 4).

For some important compounds widely reported in cereal flour, such as hexanal, the area increased during the extraction using 600 and 800 mg (Figure 4A,B). However, the decay was achieved when 1000 mg of the sample was used (Figure 4C). Comparing the signals obtained for the three sample amounts evaluated (Figure 4D), it was noticed that the signal for hexanal using 600 and 800 mg was significantly lower in comparison with the signal using 1000 mg. This reduced signal could result in the absence of decay in the area during multiple extractions. For this reason, 1000 mg was selected to optimize the MHE protocol.

A total of fifty-eight compounds were identified and their β values are shown in Table 4. β values ranging from 0.51 to 0.90 are within the limit recommended. β values below 0.4 indicate depletion of the analytes with successive extractions. On the other hand, values higher than 0.95 imply that the amount in the vial remains constant, and the area of the analyte after successive extractions is the same [38]. Therefore, the logarithm of the peak areas does not display linearity with the number of extractions. Twenty-nine percent of the identified compounds were confirmed using authentic standards (Table 4).

Nine representative compounds from different chemical classes were used to create calibration curves and perform the quantification. The method was validated, and the validation parameters are presented in Table 5. The method demonstrated adequate linearity, with R² values higher than 0.99. The limit of quantitation (LOQ) was determined as the lowest concentration that provided a detectable signal when subjected to MHE. The LOQ varied from 0.21 to 0.54 mg/kg. Intraday and interday precision were evaluated, and the results were expressed as the relative standard deviation. Precision was adequate (less than 20%), in compliance with SANTE 1312/2021 guidelines [42] (Table 5).

**Figure 5:**
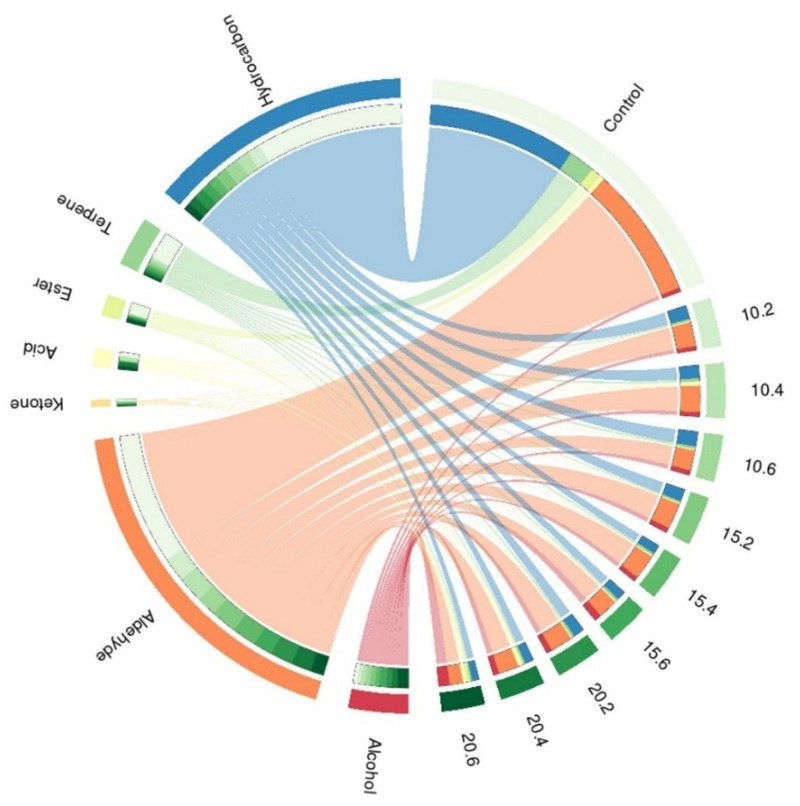
Circus plot showing the profile of volatiles quantified in IWG sample before and after germination at 10, 15, and 20 °C in 2, 4, and 6 days.

The main compounds identified in the IWG samples were aldehydes (~49%), hydrocarbons (~27%), alcohols (12%), terpenes (5%), esters (3%), organic acids (3%), and ketones (1%) (Figure 5). The ungerminated samples showed a higher concentration of aldehydes compared to the germinated ones. Among the aldehydes, hexanal and nonanal were predominant in all the samples evaluated. Increasing the germination temperature resulted in a diversification of volatiles, with an increase in acid and ester content and a reduction in aldehyde content (Figure 5).

Nonanal and hexanal have also been reported in germinated chickpea, lentil, and yellow pea flours [17]. These compounds might be formed via lipoxygenase through the oxidation of FAs. The lipoxygenase pathway is predominantly active in the green organs of plants in response to wounding [43], which may also explain its increase during germination [44]. The formation of these compounds from FAs requires one or more (1Z,4Z)-pentadienoic moieties, as found in linoleic acid and C18:2n6 [42], the main FA detected in IWG samples. The concentration of these compounds was reduced after germination compared to the ungerminated sample (control) (Figure S1—Supplementary Material). On the other hand, some minor unsaturated aldehydes such as 2-hexenal and 2-heptenal had a slight increase along with germination. However, the concentration of unsaturated compounds was more than 30-fold lower than saturated aldehydes. Moreover, the threshold of perception for 2-hexenal (17 µg/kg) is higher compared to nonanal (3 µg/kg) [33], which indicates that the increase in these compounds could have a low sensory impact on the germinated IWG.

Similar to aldehydes, the detected alcohols may be formed via the lipoxygenase pathway. 1-Hexanol, 1-octen-3-ol, and 1-pentanol were the most abundant alcohols detected in the IWG. The highest concentration for 1-hexanol (13.52 mg/kg) and 1-octen-3-ol (5.72 mg/kg) was observed after 2 days of germination at 20 °C (Table S2—Supplementary Material). Compared to the control sample, the concentration of these compounds had a slight increase over germination, as shown in Figure S2 (Supplementary Material). However, at 20 °C, the concentration of alcohol tended to decline with increased germination days. This phenomenon might be due to the booster of lipoxygenase action in the first days of germination, as has already been reported by Xu, Jin, Gu, Rao, and Chen [16], with a reduction in action along with the day of germination. Generally, alcohols are not significant contributors to the flavor of fat-containing foods due to their threshold levels being one or two orders of magnitude higher than those of the corresponding aldehydes. However, when combined with other volatiles, alcohols can enhance certain odors, such as the green or woody notes characteristic of these compounds [33].

4-Hydroxy-4-methyl-2-pentanone was the main ketone detected in the IWG samples, with concentrations ranging from 0.93 to 2.91 mg/kg (Table S2—Supplementary Material). The highest concentration was detected at 10 °C after 2 days of germination, with a continuous decrease in concentration as germination progressed. Interestingly, 4-hydroxy-4-methyl-2-pentanone has been associated with antimicrobial, insecticidal, and phytotoxic activities, suggesting its role as a defensive compound against pathogens, insect herbivores, and competitive plants. Moreover, it has shown phytotoxic effects, being capable of inhibiting the germination of other seeds, such as corn and wheat. However, its function in plants remains unclear [45].

3-Octen-2-one was also detected in the germinated samples at concentrations ranging from 1.09 to 0.6 mg/kg. This compound is associated with the LOX pathways and has been reported in other studies using germinated flours of chickpeas, lentils, and yellow peas [16,17]. In this study, a minimal variation in 3-octen-2-one concentration was observed when the samples were germinated at 15 and 20 °C for 6 days. A similar behavior was observed by Lan, Wang, Wang, Zhang, Song, Zhao, Yang, and Liu [10] after 48 h of quinoa germination, which could be attributed to the loss of enzyme activity due to substrate reduction or denaturation [11].

Regarding the hydrocarbons, the main compounds detected were styrene and 3-methylpentadecane. Styrene was first identified in storax [46], a resin used in perfumery, and it is responsible for the sweet balsamic characteristic of some plants [47]. Conversely, reports on the odor or incidence of 3-methylpentadecane are scarce, but few studies report its incidence in vegetables [48,49]. The concentrations of these compounds significantly reduced after germination, from 708.56 to 18.71 mg/kg for styrene and from 48.02 to 2.73 mg/kg for 3-methylpentadecane. It was also observed for esters, organic acids, and terpenes. It might be associated with the oxidation to generate energy during germination in a process similar to the β-oxidation of lipids [50]. The same behavior was also observed in germinated faba beans [19].

**Figure 6:**
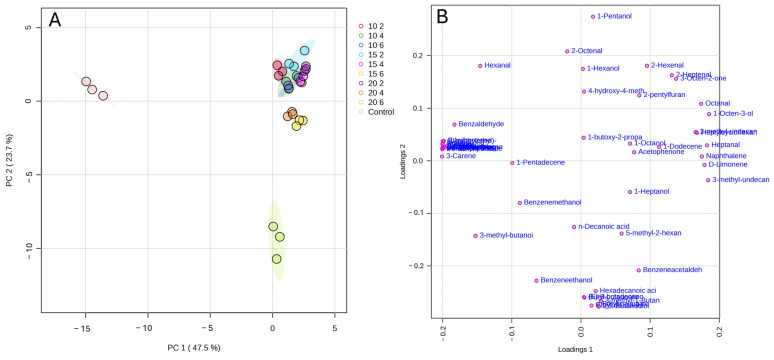
Principal component analysis with score plots (**A**) and loadings (**B**) for volatiles from IWG samples.

A principal component analysis (Figure 6A,B) was performed using the volatile compound concentrations of the IWG samples. The two principal components (PC1 and PC2) explained more than 71% of the data. The impact of hexanal (loading 1, −0.14568) and nonanal (loading 1, −0.20006) was observed by separating the ungerminated samples from the germinated ones. Additionally, esters and organic acids such as ethyl cis,cis-9,12-octadecadienoate (loading 2, −0.27537) and ethyl palmitate (loading 2, 0.27298) discriminated the samples germinated at 20 °C after 6 days.

Volatile compounds and FAs were evaluated using Pearson correlation (Table S3—Supplementary Material). The results showed a positive and significant correlation between the unsaturated FAs and some volatile compounds. Correlation values higher than 0.65 were found for C18:1 cis and hexanal (*p* < 0.005) and C18:2 n-6 and 2-nonenal (*p* < 0.005).

Overall, germination can be used to improve the volatile profile of seeds [9]. In this study, many volatiles responsible for off-odors decreased as germination progressed. At 10 °C and 15 °C, germination time had a more significant effect on the reduction of these compounds than temperature. However, at 20 °C, the concentration of some esters and organic acids, such as butyl butanoate, n-decanoic acid, and ethyl palmitate, significantly increased with the germination time, suggesting that a fermentation process could have occurred.

## 3. Materials and Methods

### 3.1. Samples

Intermediate wheatgrass was steeped for 24 h using 500 mL of deionized water to achieve a 42–45% moisture content. One hundred and fifty grams of grains was germinated in plastic trays at 10, 15, or 20 °C for 2, 4, or 6 days (Figure 1). The volume of water (500 mL) was replaced every 24 h followed by stirring. After germination, the grains were dried at 50 °C (16 h) and the rootles were separated. The grains were then milled (UDY Mill; 0.5 mm screen) and stored at 4 °C until analysis. A flour made with a non-germinated IWG sample was used as a control.

### 3.2. Chemicals and Reagents

Hydrochloric acid (HCl), toluene, methanol, ethylenediaminetetraacetic acid disodium salt dihydrate (EDTA2Na), butylated hydroxytoluene (BHT), and HPLC-grade hexane and chloroform were purchased from Sigma (St Louis, MO, USA). Triheptadecanoin (Tri-C17:0) was purchased from Cayman (Ann Arbor, MI, USA). FAME 37 mix standard was purchased from Supelco (Bellefonte, PA, USA).

Volatile standards including hexanal (95%), 4-hydroxy-4-methyl-2-pentanone (99%), cumene (99%), 2-heptanone (99%), trans-2-heptenal (95%), benzaldehyde (99%), 1-heptanol (99%), 1-octen-3-ol (98%), octanal (98%), 2-ethyl-1-hexanol (99%), limonene (99%), benzyl alcohol (99%), trans-2-octenal (96%), acetophenone (99%), nonanal (99%), 2-phenylethanol (99%), terpinen-4-ol (95%), naphthalene (99%), dodecane (99%), decanal (98%), nonanoic acid (99%), tetradecane (99%), 2-heptanol (98%), 1,3-dimethylbenzene (98%), and n-alkanes were purchased from Sigma (St Louis, MO, USA). γ-hexalactone (98%) was purchased from Thermo Scientific Chemicals (Rockwood, TN, USA). 3-methyl-1-butanol was purchased from EP Scientific Products (Miami, OK, USA).

### 3.3. FA Profile

FAs from IWG samples were extracted using the Folch method and derivatized according to the protocol proposed by Dias et al. [51] with some modifications. Each sample was prepared in triplicate (*n* = 3). Briefly, 112 mg of IWG (~4 mg of lipid) flour was weighed into glass tubes. Then, 3.0 mL of chloroform/methanol (2:1 *v*/*v*) with 0.002% (*w*/*v*) BHT was added, followed by vortexing for 20 s. Next, 750 µL of 0.9% (*w*/*v*) NaCl solution containing 1 mM EDTA2Na was added, and the mixture was vortexed again. The samples were centrifuged at 740× *g* for 10 min at 0 °C. The lower organic phase was transferred to another tube, and the remaining sample was re-extracted using 2 mL of chloroform, followed by vortexing and centrifugation at 740× *g* for 10 min at 0 °C. The organic phases were combined, dried under nitrogen flux, and resuspended in 200 µL of chloroform/methanol mixture (2:1 *v*/*v*).

For the derivatization step, the lipid extract was spiked with 0.6 mg of triheptadecanoin (Tri-C17:0, 15 mg/mL), followed by the addition of 0.4 mL of toluene and vortexed for 20 s. Afterwards, 3 mL of methanol and 0.6 mL of 8% concentrated HCl (37%) in methanol were added, followed by another 20 s of vortexing. The samples were heated for 1 h at 90 °C. After cooling down, 1 mL of water and 1 mL of hexane were added, followed by additional mixing. The samples were left to rest to allow phase separation. Nine hundred microliters of the hexane layer was transferred to 2.0 mL centrifuge tubes containing 450 µL of ultrapure water, vortexed, and centrifuged for 2 min at 15,871× *g*. The upper hexane layer was transferred to a new tube, dried under nitrogen, and re-suspended in 100 µL of hexane.

The samples were analyzed using a gas chromatograph coupled to a flame ionization detector (GC-FID, Agilent 6890N, Agilent Technologies, Santa Clara, CA, USA). One microliter of each sample was injected in split mode (1:30). The separation was performed using an FFAP column (30 m × 0.25 mm × 0.25 µm). The injector was maintained at 240 °C and the detector at 300 °C. The oven temperature started at 50 °C for 2 min, increased to 180 °C at a rate of 10 °C/min, followed by a ramp to 240 °C at 5 °C/min, and was held at 240 °C for 13 min. Hydrogen at a rate of 1 mL/min was used as the carrier gas. Peak identification was performed by comparing the FAME 37 standards’ retention time with those obtained in the samples under the same analytical conditions. Relative quantification was performed using internal standards.

### 3.4. Volatile Profile

#### 3.4.1. SPME Fiber Coating Selection

Data on the quantitative profile of IWG volatiles have not yet been reported. Therefore, the optimal conditions, including the fiber coating material, for extracting volatiles from IWG were evaluated. Three SPME fiber coatings were tested, aiming to select the one with the highest volatile extractability. The coatings tested were PDMS (polydimethylsiloxane, 1 cm, 7 µm), DVB/PDMS (divinylbenzene/polydimethylsiloxane, 1 cm, 65 µm), and DVB/CAR/PDMS (divinylbenzene/carboxen/polydimethylsiloxane, 1 cm, 80 µm). Before use, all fibers were properly conditioned according to the manufacturer’s recommendation (Supelco, Bellefonte, PA, USA).

The fibers were exposed to the HS of the samples (600 mg) under the same conditions for comparison purposes: pre-incubation for 10 min at 50 °C under stirring at 300 rpm, followed by extraction at the same temperature for 60 min [52] using a PAL System RSI 120. After extraction, the fibers were immediately introduced into the GC injector for the desorption of the analytes at 280 °C, in splitless mode, for 1 min. All the extractions were performed in triplicate, and the fiber with the largest total area of the chromatogram was selected for the further steps of extraction optimization.

#### 3.4.2. Optimization of SPME Extraction Conditions

The use of a multivariate statistical approach allows us, with a reduced number of experiments, to determine the conditions to analyze with optimized performance parameters [53]. For this reason, after selecting the fiber, we optimized the main extraction parameters such as time and temperature using a 2^2^ CCRD with four axial points (α = 1.41) and three repetitions in the central point (0), totaling 12 experiments. Extraction temperatures (X1) varying from 35 °C to 65 °C and extraction time (X2) between 30 min and 60 min were evaluated (Table 6). These variables were selected since they are described as determinants for the process of extracting volatiles from flour and other matrices [52,54,55]. Total area was used as a response. After optimization, volatile extractions were performed in triplicate, under optimal extraction conditions.

#### 3.4.3. Identification and Quantification of Volatiles from IWG by HS-SPME-GC/MS

The determination of the volatile compounds was carried out using an Agilent 6890 gas chromatograph equipped with a PAL RSI 120 autosampler and coupled to an Agilent 5973 single-quadrupole detector (Agilent Technologies, Palo Alto, CA, USA). A DB-5ms Ultra Inert column (30 m × 250 μm × 0.25 μm) was used. The inlet was set at 280 °C and in spitless mode. Helium at 1.2 mL/min was used as carrier gas. The oven temperature began at 40 °C followed by a ramp to 220 °C at 4 °C/min, which was held for 5 min, totaling 50 min of chromatography. The quadrupole, MS ion source, and transfer line were set at 150, 230, and 250 °C, respectively. Electron impact mass spectra were recorded at 70 eV ionization energy in scan mode (*m*/*z* 40–400).

Volatile compound identification was conducted utilizing mass spectrometry (MS) with Agilent MassHunter Unknown software and the NIST 17 MS library. Only compounds exhibiting a signal-to-noise ratio greater than 3 and a match score higher than 80 were considered. To confirm the identity of the compounds, the Van den Dool and Kratz programmed temperature retention indexes (RIs) were calculated by injecting a solution of n-alkanes (C7–C20) (Supelco, Alltech, PA, USA) into the GC-MS under the same conditions. Only compounds with a maximum RI variation of 30 units were considered. When available, positive identification was performed by comparing the retention time of the compound of interest with that of authentic standards.

The quantification of the compounds was performed by MHE. The MHE allows for the quantification of compounds by SPME by converting an equilibrium technique to an exhaustive technique by carrying out several extractions of the same sample. The sum of the instrumental response from each step of HS extraction corresponds to the total area (AT) of the compound, and can be obtained by Equation (1):

(1) $$A_{T}=\sum_{i=1}^{i\to \infty }A_{i}=\frac{A_{1}}{1-e^{-q}}=\frac{A_{1}}{1-\beta }$$

where A_T_ is the total peak area, A_1_ is the peak area of the first extraction, and q is a constant describing the exponential decay associated with β [20].

The constant β is determined by the slope of the regression curve plotted using the logarithm of the areas of the individual peaks and the number of extractions, as described by Equation (2):

(2) $$\ln A_{i}=\ln A_{1}+(i-1)\cdot \ln\beta$$

where A_i_ is the area obtained in the i^th^ extraction. This linear equation is represented as y = ax + b, where ln A_1_ is the y-axis intercept and lnβ is the slope.

Afterward, external calibration curves can be used for accurate quantitation of the compound in the sample, using only the analyte response after the first extraction step (A_1_) [13,20].

For MHE, the amount of sample and number of extractions should be carefully evaluated. Sample size in MHE is crucial since small samples can lose significant mass between extractions and do not show exponential decay while large samples can cause HS saturation, which also affects quantification [39]. For this evaluation, six extractions were performed with sample sizes of 600, 800, and 1000 mg.

After sample size optimization, the IWG flours were weighted in 20 mL glass vials and subjected to six consecutive extractions. The concentration of each volatile was determined using external calibration curves. Five microliters of calibration solution prepared in dibutyl phthalate was subjected to multiple HS analyses under the same conditions. Decanal was used as the internal standard and was pre-loaded into the fiber before each extraction. This approach minimizes errors associated with adding a standard to the sample and enables monitoring of the reliability and efficiency of the SPME during use [56].

Validation parameters such as limits of detection and quantification, linearity, and precision were determined. The limit of detection was determined as three times the noise divided by the slope of the analytical curve [38]. The limit of quantification was defined as the lowest concentration that produced a signal decay during multiple extractions. Intraday and interday precision were determined from three different points on the calibration curves (LOQ, midpoint, and highest point of the calibration curve). Intraday precision was evaluated by analyzing the prepared curve 10 times in a row on the same day, while interday precision was assessed by analyzing the samples on 3 consecutive days. Precision was expressed as the relative standard deviation of the sample replicates. Analytical curves were plotted using total area versus concentration. The concentrations in the sample were estimated using the term β and the total area was calculated using the first extraction. GC-MS analysis was performed as described above.

### 3.5. Statistical Analysis

All measurements were performed in triplicate and values were expressed as mean ± SD. The significance of the model proposed by the multivariate optimization was assessed using ANOVA (with α = 0.05), considering the coefficient of determination (*R*²) and the lack of fit. The FA and volatile concentration were evaluated using ANOVA followed by Tukey’s test with a 95% confidence level. Model evaluation and ANOVA were performed using Statistica 14 software (Statsoft, Tulsa, OK, USA). Principal component analysis (PCA) was generated to better visualize the differences between variables and track trends between the samples. Circus plot graphs were generated with Omicstudio https://www.omicstudio.cn, accessed on 10 July 2024). The PCA was performed using the concentration of each identified volatile compound. The data were auto-scaled and analyzed using MetaboAnalyst 5.0 https://www.metaboanalyst.ca, accessed on 12 July 2024.

## 4. Conclusions

For the first time, the effects of germination on the volatile and FA profile of IWG were reported. An MHE method was developed with the aid of multivariate evaluation. More than 50 compounds were identified and quantified, the majority of which were generated through the oxidation of linoleic acid (C18:2n6). Overall, germination at 10 °C and 15 °C reduced the concentration of aldehydes, ketones, and alcohols. However, at 20 °C, the concentration of organic acids and esters increased, suggesting the formation of new off-notes via fermentation. The days of germination showed a greater effect on the reduction of FAs than the temperature. Germinating at 15 °C for 6 days resulted in the most effective reduction of aldehydes and alcohols in IWG, including nonanal, hexanal, 1-pentanol, and 1-hexanol. Pearson correlation analysis revealed a significant association between the concentrations of these compounds and the content of C18:2n6. This study sheds new light on the effects of critical germination parameters, including time and temperature, on IWG flour. Future applications of germinated IWG present exciting opportunities to diversify the existing range of morning cereals, pastas, beers, puffs, and more. The food industry can leverage this to drive innovation and promote sustainability by creating new products that incorporate germinated IWG. However, the success of these products hinges on comprehensive sensory evaluations. It is crucial to understand how the reduction of lipid oxidation products influences the flavor and aroma of the final products, ensuring that they meet consumer expectations for taste and quality. Our findings pave the way for enhanced utilization and broaden the potential applications of this sustainable crop.

## Figures and Tables

**Table 1:** Concentration of FAs (mg/g) in IWG samples after germination by 2, 4, and 6 days at 10, 15, and 20 °C.

	C14:0	C15:0	C16:0	C16:1	C18:0	C18:1cis	C18:2n-6	C18:3n-6	C18:3n-3	C20:0	C20:1n-9
Control	0.03 ± 0.02 ^A^	0.02 ± 0.00 ^AB^	2.6 ± 0.4 ^A^	0.03 ± 0.00 ^C^	0.24 ± 0.16 ^A^	3.74 ± 0.44 ^A^	13.76 ± 1.58 ^A^	0.05 ± 0.02 ^AB^	1.12 ± 0.10 ^AB^	0.02 ± 0.00 ^A^	0.17 ± 0.01 ^AB^
10-2	0.02 ± 0.00 ^A^	0.02 ± 0.00 ^B^	2.13 ± 0.09 ^AB^	0.03 ± 0.01 ^C^	0.16 ± 0.07 ^A^	2.97 ± 0.25 ^ABC^	10.92 ± 0.77 ^AB^	0.03 ± 0.00 ^AB^	1.03 ± 0.05 ^B^	0.02 ± 0.00 ^A^	0.15 ± 0.01 ^AB^
10-4	0.03 ± 0.03 ^A^	0.02 ± 0 ^AB^	2.59 ± 0.23 ^A^	0.05 ± 0.01 ^ABC^	0.26 ± 0.17 ^A^	3.63 ± 0.46 ^AB^	13.17 ± 1.99 ^AB^	0.04 ± 0.00 ^AB^	1.3 ± 0.19 ^A^	0.02 ± 0.02 ^A^	0.19 ± 0.03 ^A^
10-6	0.02 ± 0.00 ^A^	0.02 ± 0.00 ^B^	1.96 ± 0.12 ^C^	0.04 ± 0.00 ^BC^	0.17 ± 0.06 ^A^	2.67 ± 0.09 ^C^	10.28 ± 0.69 ^B^	0.03 ± 0.00 ^B^	1.06 ± 0.06 ^AB^	0.02 ± 0.00 ^A^	0.13 ± 0.01 ^C^
15-2	0.02 ± 0.00 ^A^	0.02 ± 0.00 ^AB^	2.3 ± 0.13 ^AB^	0.05 ± 0.01 ^BC^	0.14 ± 0.01 ^A^	3.29 ± 0.23 ^ABC^	11.82 ± 0.57 ^AB^	0.06 ± 0.01 ^A^	1.08 ± 0.06 ^AB^	0.02 ± 0.00 ^A^	0.17 ± 0.02 ^AB^
15-4	0.02 ± 0.01 ^A^	0.02 ± 0.00 ^AB^	2.26 ± 0.12 ^AB^	0.05 ± 0.00 ^ABC^	0.24 ± 0.12 ^A^	3.23 ± 0.17 ^ABC^	11.79 ± 0.69 ^AB^	0.05 ± 0.01 ^AB^	1.13 ± 0.06 ^AB^	0.03 ± 0.00 ^A^	0.18 ± 0.02 ^AB^
15-6	0.02 ± 0.00 ^A^	0.02 ± 0.00 ^AB^	2.05 ± 0.10 ^C^	0.06 ± 0.01 ^ABC^	0.15 ± 0.01 ^A^	2.71 ± 0.18 ^C^	10.34 ± 0.67 ^B^	0.04 ± 0.01 ^AB^	0.98 ± 0.05 ^B^	0.02 ± 0.00 ^A^	0.14 ± 0.01 ^AB^
20-2	0.02 ± 0.00 ^A^	0.02 ± 0.00 ^AB^	2.22 ± 0.12 ^AB^	0.05 ± 0.00 ^ABC^	0.18 ± 0.07 ^A^	3.13 ± 0.19 ^ABC^	11.23 ± 0.73 ^AB^	0.04 ± 0.00 ^AB^	0.99 ± 0.07 ^B^	0.03 ± 0.00 ^A^	0.17 ± 0.01 ^AB^
20-4	0.03 ± 0.00 ^A^	0.02 ± 0.00 ^A^	2.23 ± 0.12 ^AB^	0.07 ± 0.02 ^AB^	0.21 ± 0.02 ^A^	2.9 ± 0.07 ^BC^	10.86 ± 0.57 ^AB^	0.04 ± 0.00 ^AB^	0.95 ± 0.04 ^B^	0.03 ± 0.00 ^A^	0.16 ± 0.00 ^AB^
20-6	0.03 ± 0.01 ^A^	0.02 ± 0.00 ^A^	2.17 ± 0.17 ^AB^	0.08 ± 0.02 ^A^	0.24 ± 0.05 ^A^	2.88 ± 0.31 ^BC^	10.9 ± 0.95 ^AB^	0.03 ± 0.01 ^B^	0.95 ± 0.09 ^B^	0.03 ± 0.00 ^A^	0.15 ± 0.02 ^AB^

Different letters indicate statistically significant differences by Tukey’s test at *p* < 0.05. 10-2, -4, and -6: 10 °C and 2-, 4- and 6-day germination, respectively.

**Table 2:** Experimental conditions (coded/real values) and response for the optimization of the extraction conditions of volatile compounds from IWG flour by HS-SPME.

Trial	Temp (°C)	Time (min)	Total Area
1	−1 (39.40)	−1 (34.36)	669,149,921
2	1 (60.60)	−1 (34.36)	733,292,935
3	−1 (39.40)	1 (55.64)	750,239,779
4	1 (60.60)	1 (55.64)	845,258,191
5	−1.41 (35.00)	0 (45.00)	656,168,788
6	1.41 (65.00)	0 (45.00)	846,707,555
7	0 (50.00)	−1.41 (30.00)	639,981,632
8	0 (50.00)	1.41 (60.00)	808,548,691
9	0 (50.00)	0 (45.00)	711,222,561
10	0 (50.00)	0 (45.00)	734,532,059
11	0 (50.00)	0 (45.00)	672,019,319
12	0 (50.00)	0 (45.00)	669,149,921

**Table 3:** Analysis of variance (ANOVA) including models and R^2^ for the extraction of volatile-form IWG by HS-SPME.

Source of Variation	Sum of Squares	Degrees of Freedom	Mean of Squares (MS)	F Cal	F Tab
Regression (R)	5.03 × 10^16^	5	1.01 × 10^16^	12.39	5.05
Residue (r)	4.06 × 10^15^	5	8.12 × 10^14^		
Lack of fit	2.06 × 10^15^	3	6.88 × 10^14^	0.69	19.6
Pure error	1.97 × 10^15^	2	9.99 × 10^14^		
Total	5.44 × 10^16^	10			

**Table 4:** Volatile compounds identified by HS-SPME and GC-MS in IWG samples under optimal extraction conditions.

RT	Compounds	Odor Descriptors ^A^	RI	RI Tab	Δ	Identification	β
	Alcohols						
3.11	3-methyl-1-butanol	Malty	776	735	24	RI, MS, STD	0.78
3.6	1-pentanol	Fruity, ethereal	795	780	15	RI, MS	0.78
4.08	2,3-butanediol	Butter-like	813	819	−6	RI, MS	0.78
5.86	1-hexanol	Grassy	883	880	3	RI, MS	0.78
6.73	2-heptanol	Coconut-like	916	900	16	RI, MS, STD	0.90
7.94	1-butoxy-2-propanol	Ether-like ^B^	953	947	6	RI, MS	0.78
8.96	1-heptanol	Fruity, soapy	984	975	9	RI, MS, STD	0.78
9.3	1-octen-3-ol	Mushroom-like	994	986	8	RI, MS, STD	0.72
11.15	Benzyl alcohol	Bitter almond-like	1047	1042	5	RI, MS	0.78
12.37	2-octen-1-ol	Soapy	1081	1067	14	RI, MS	0.51
12.51	1-octanol	Soapy	1085	1076	9	RI, MS	0.78
13.94	Phenylethyl alcohol	Floral	1124	1121	3	RI, MS	0.78
16.34	p-menthan-3-ol	Peppermint-like	1191	1170	21	RI, MS	0.78
	Aldehydes						
4.22	Hexanal	Grassy	819	817	2	RI, MS, STD	0.89
5.44	2-hexenal	Green apple-like	866	854	12	RI, MS	0.89
6.79	Heptanal	Fatty	918	907	11	RI, MS	0.89
8.51	2-heptenal	Green apple-like	970	978	−8	RI, MS, STD	0.51
8.65	Benzaldehyde	Almond-like	974	965	9	RI, MS, STD	0.51
10.11	Octanal	Green	1018	1007	11	RI, MS, STD	0.89
11.49	Phenylacetaldehyde	Floral	1056	1053	3	RI, MS	0.89
12.04	2-octenal	Fatty	1072	1061	11	RI, MS, STD	0.51
13.74	Nonanal	Soapy	1119	1108	11	RI, MS, STD	0.89
15.7	2-nonenal	Fatty	1173	1171	2	RI, MS	0.51
	Ketones						
5.11	4-hydroxy-4-methyl-2-pentanone	Mint-like ^B^	854	850	4	RI, MS, STD	0.54
5.5	5-methyl-2-hexanone	Ethereal	869	857	12	RI, MS	0.84
6.41	2-heptanone	Fruity	904	898	6	RI, MS, STD	0.54
6.98	γ-butyrolactone	Sweety	923	922	1	RI, MS	0.54
11.3	3-octen-2-one	Floral, spicy	1051	1046	5	RI, MS	0.54
11.73	γ-caprolactone	Fruity	1063	1055	8	RI, MS, STD	0.54
12.25	Acetophenone	Foxy	1077	1073	4	RI, MS, STD	0.54
12.42	3,5-octadien-2-one	Fatty, fruity	1082	1093	11	RI, MS	0.54
22.51	γ-nonalactone	Coconut-like	1369	1368	1	RI, MS	0.54
	Acids						
5.28	3-methyl-butanoic acid	Sweaty	860	834	26	RI, MS	0.78
6.24	Pentanoic acid	Sweaty, fruity	897	875	22	RI, MS	0.90
22.94	Decanoic acid	Soapy, musty	1382	1380	2	RI, MS	0.90
39.51	Hexadecanoic acid	Waxy ^B^	1967	1960	7	RI, MS	0.90
	Esters						
9.86	Butyl butanoate	Sweet, fruity	1011	993	18	RI, MS	0.64
20.04	Bornyl acetate	Pine-like	1296	1291	5	RI, MS	0.64
40.37	Ethyl palmitate	Waxy ^B^	2001	1996	5	RI, MS	0.90
44.18	Ethyl cis,cis-9,12-octadecadienoate	-	2166	2159	7	RI, MS	0.90
44.34	(E)-9-octadecenoic acid ethyl ester	-	2173	2174	−1	RI, MS	0.90
	Terpenes						
7.74	2-pinene	Resin-like	947	939	8	RI, MS	0.90
8.39	Dehydrosabinene	-	966	957	9	RI, MS	0.90
10.25	3-carene	Terpene-like	1022	1017	5	RI, MS	0.90
10.82	p-cymene	Petrol-like	1038	1030	8	RI, MS	0.90
10.98	Limonene	Citrus-like	1042	1032	10	RI, MS, STD	0.88
24.91	trans-α-bergamotene	Woody-like	1444	1441	3	RI, MS	0.88
	Hydrocarbons and others						
5.63	Ethylbenzene	Petrol-like ^B^	874	868	6	RI, MS	0.90
6.47	Styrene	Balsam-like	906	895	11	RI, MS	0.90
7.42	(1-methylethyl)-benzene	Petrol-like ^B^	937	929	8	RI, MS, STD	0.90
9.63	2-pentylfuran	Vegetable-like	1004	1010	−6	RI, MS	0.90
15.89	2-methyl-undecane	-	1178	1167	11	RI, MS	0.64
16.11	3-methyl-undecane	-	1184	1171	13	RI, MS	0.64
16.49	Naphthalene	Smoky	1195	1196	−1	RI, MS, STD	0.64
16.86	1-dodecene	-	1205	1192	13	RI, MS	0.64
22.06	Heptylcyclohexane	-	1356	1345	11	RI, MS	0.64
26.83	1-pentadecene	-	1504	1494	10	RI, MS	0.90
29.17	3-methyl-pentadecane	-	1582	1566	16	RI, MS	0.90

RT: retention time, RI: retention index calculated, RI tab: retention index reported in the literature, MS: mass spectra, STD: identification using standard. Odor descriptors were collected from TUM (A) odorant database [40] or PubChem [41] (B).

**Table 5:** Validation parameters for the MHE-SPME and GC-MS method.

Compounds	LOQ (mg·kg^−1^)	Linear Range (mg·kg^−1^)	Equation	R^2^	Precision	
					Intraday	Interday
2-Heptanol	0.54	0.54–16.0	y = 920,915x − 219,377	0.993	18.0	14.1
Cumene	0.49	0.49–14.79	y = 46,063x + 155,054	0.999	5.9	6.0
1-Octen-3-ol	0.48	0.48–14.86	y = 700,623x − 223,938	0.991	15.1	9.9
Octanal	0.45	0.45–17.66	y = 46,063x + 155,054	0.999	7.9	5.3
Limonene	0.21	0.21–15.06	y = 466,801x + 127,215	0.991	12.5	9.0
Trans-2-Octenal	0.40	0.40–15.35	y = 140,548x + 31,426	0.992	14.4	8.8
Acetophenone	0.41	0.41–14.76	y = 671,632x + 103,442	0.998	13.1	7.4
Naphthalene	0.44	0.44–14.92	y = 1,730,517x − 194,167	0.992	8.1	4.8

LOQ: limit of quantification, LOD: limit of detection.

**Table 6:** Factors and levels applied to optimize the extraction conditions of volatiles from IWG samples by HS-SPME.

Variables	Levels				
	−1.41	−1	0	1	1.41
Temperature (°C) (X1)	35.00	39.40	50.00	60.60	65.00
Time (min) (X2)	30.00	34.36	45.00	55.64	60.00

## Data Availability

All data generated or analyzed during this study are included in the published article.

## Supplementary Materials

The following supporting information can be downloaded at https://www.mdpi.com/article/10.3390/molecules29174268/s1, Table S1: Fatty acid profile (%) in IWG samples germinated at 10, 15 and 20 °C and 2, 4 and 6 days of germination; Table S2: Compounds quantified (mg.kg-1) in IWG sample without germination (control) and after germination by 2, 4 and 6 days at 10, 15, and 20 °C; Figure S1: Changes in the concentration of hexanal (A), nonanal (B), 2-hexenal (C), and 2-heptenal (D) during germination of IWG for 2, 4 and 6 days at 10, 15, and 20 °C; Figure S2: Variation of 1-pentanol (A), 1-hexanol (B), and 1-octenol (C) during the germination of IWG for 2, 4, and 6 days at 10 °C, 15 °C, and 20 °C; Table S3: Correlation between unsaturated fatty acids and volatiles compounds quantified in IWG samples.
