# Supplementary material for: Unraveling the Impacts of Germination on the Volatile and Fatty Acid Profile of Intermediate Wheatgrass (Thinopyrum intermedium) Seeds
Source: Molecules. 2024 Sep 9;29(17):4268. doi: 10.3390/molecules29174268 (PMC11397152; doi:10.3390/molecules29174268)
Supplement: Supplementary file 1 [file molecules-29-04268-s001.zip › molecules-3188835-supplementary.pdf]

Supplementary material

Unraveling the impacts of germination on the volatile and fatty acid profile of intermediate wheatgrass (*Thinopyrum intermedium*) seeds

Wellington S. Oliveira, Qianqian Chen, Dana Edleman, George A. Annor, and Fernanda F. G. Dias*

Table S1. Fatty acid profile (%) in IWG samples germinated at 10, 15 and 20 °C and 2, 4 and 6 days of germination.

Fatty acid	Code	Control 1	10_2	10_4	10_6	15_2	15_4	15_6	20_2	20_4	20_6
Myristic Acid	C14:0	0.1	0.1	0.2	0.1	0.1	0.1	0.1	0.1	0.1	0.2
Pentadecylic acid	C15:0	0.1	0.1	0.1	0.1	0.1	0.1	0.1	0.1	0.1	0.1
Palmitic Acid	C16:0	11.9	12.2	12.2	12.0	12.1	11.9	12.4	12.3	12.6	12.5
Palmitoleic Acid	C16:1	0.1	0.2	0.3	0.2	0.2	0.3	0.3	0.3	0.3	0.4
Stearic Acid	C18:0	1.0	0.9	1.3	1.1	0.8	1.2	0.9	1.0	1.3	1.4
Oleic Acid +Elaidic acid	C18:1 cis	17.2	17.0	17.1	16.3	17.3	17.0	16.4	17.3	16.9	16.4
Linoleic Acid	C18:2 n-6	63.2	62.4	61.7	62.6	62.4	62.0	62.5	62.1	61.9	62.3
Gamma-linolenic acid	C18:3 n-6	0.3	0.2	0.2	0.2	0.3	0.3	0.3	0.2	0.2	0.2
Linolenic Acid	C18:3 n-3	5.1	5.9	6.1	6.5	5.7	5.9	5.9	5.5	5.4	5.4
Arachidic acid	C20:0	0.1	0.1	0.1	0.1	0.1	0.2	0.1	0.1	0.1	0.2
Gondoic Acid/11-eicosanoic acid	C20:1 n-9	0.8	0.9	0.9	0.8	0.9	0.9	0.9	1.0	0.9	0.9

Table S2. Compounds quantified (mg.kg^{-1}) in IWG sample without germination (control) and after germination by 2, 4 and 6 days at 10, 15, and 20 ºC

Compound	Analytical curve	Control	10 2	10 4	10 6	15 2	15 4	15 6	20 2	20 4	20 6
3-methyl-1-Butanol	1	<LOQ	<LOQ	<LOQ	0.72 ± 0.16 ^{CD}	<LOQ	0.63 ± 0.04 ^{D}	1.58 ± 0.15 ^{B}	<LOQ	1 ± 0.15 ^{C}	2.39 ± 0.27 ^{A}
1-Pentanol	1	2.77 ± 0.19 ^{BCD}	2.8 ± 0.32 ^{BCD}	3.27 ± 0.39 ^{AB}	2.99 ± 0.19 ^{ABCD}	3.65 ± 0.25 ^{A}	3.06 ± 0.2 ^{ABC}	2.57 ± 0.21 ^{CD}	3.38 ± 0.21 ^{AB}	2.37 ± 0.04 ^{D}	<LOQ
2,3-Butanediol	3	0.69 ± 0.06 ^{D}	0.77 ± 0.12 ^{D}	1.13 ± 0.23 ^{D}	1.36 ± 0.16 ^{D}	1.31 ± 0.11 ^{D}	2.22 ± 0.41 ^{D}	16.7 ± 0.43 ^{B}	6.39 ± 0.1 ^{C}	8.91 ± 0.94 ^{C}	46.77 ± 4.08 ^{A}
Hexanal	4	94.75 ± 4.6 ^{A}	51.57 ± 1.46 ^{C}	49.17 ± 2.43 ^{C}	38.28 ± 4.46 ^{DE}	64 ± 7.53 ^{B}	44.85 ± 3.32 ^{CD}	28.77 ± 1.52 ^{E}	49.2 ± 1.77 ^{C}	32.19 ± 0.75 ^{E}	13.45 ± 0.74 ^{F}
4-hydroxy-4-methyl-2-Pentanone	7	1.57 ± 0.22 ^{CD}	2.91 ± 0.13 ^{A}	1.29 ± 0.11 ^{DEF}	1.31 ± 0.28 ^{DEF}	1.68 ± 0.19 ^{BCD}	1.49 ± 0.11 ^{CDE}	2.05 ± 0.06 ^{B}	1.74 ± 0.04 ^{BC}	1.09 ± 0.01 ^{EF}	0.93 ± 0.07 ^{F}
3-methyl-butanoic acid	2	<LOQ	<LOQ	<LOQ	<LOQ	<LOQ	<LOQ	<LOQ	<LOQ	1.35 ± 0.16 ^{A}	1.51 ± 0.1 ^{A}
2-Hexenal	4	5.31 ± 0.43 ^{E}	14.51 ± 2.56 ^{ABC}	11.6 ± 0.17 ^{BCD}	10.42 ± 2.09 ^{D}	15.64 ± 1.82 ^{A}	9.97 ± 1.26 ^{D}	10.79 ± 1.25 ^{CD}	10.05 ± 0.4 ^{D}	14.93 ± 0.14 ^{AB}	3.07 ± 0.05 ^{E}
5-methyl-2-hexanone	7	<LOQ	<LOQ	<LOQ	<LOQ	<LOQ	<LOQ	0.85 ± 0.04 ^{AB}	0.88 ± 0.02 ^{A}	0.81 ± 0.02 ^{BC}	0.77 ± 0.02 ^{C}
Ethylbenzene	8	10.89 ± 1.77 ^{A}	1.22 ± 0.16 ^{B}	1.39 ± 0.22 ^{B}	1.3 ± 0.25 ^{B}	1.15 ± 0.15 ^{B}	<LOQ	<LOQ	1.19 ± 0.08 ^{B}	<LOQ	<LOQ
1-Hexanol	1	11.88 ± 1.05 ^{AB}	10.21 ± 1.19 ^{BC}	10.1 ± 0.45 ^{BC}	11.97 ± 0.57 ^{AB}	12.88 ± 2.06 ^{AB}	11.35 ± 0.91 ^{AB}	12.44 ± 0.61 ^{AB}	13.52 ± 0.51 ^{A}	10.98 ± 0.15 ^{ABC}	8.56 ± 0.7 ^{C}
Pentanoic acid	2	7.61 ± 0 ^{A}	<LOQ	<LOQ	<LOQ	<LOQ	<LOQ	<LOQ	<LOQ	1.08 ± 0.19 ^{B}	0.77 ± 0 ^{C}
Styrene	8	708.55 ± 59.62 ^{A}	38.05 ± 6.44 ^{B}	37.29 ± 5.63 ^{B}	42.13 ± 1.48 ^{B}	39.69 ± 9.75 ^{B}	25.44 ± 2.95 ^{B}	26.66 ± 4.05 ^{B}	28.86 ± 1.06 ^{B}	22.48 ± 0.32 ^{B}	18.71 ± 0.9 ^{B}
Heptanal	4	<LOQ	11.31 ± 1.87 ^{A}	10.22 ± 1.6 ^{A}	12.79 ± 1.47 ^{A}	13.68 ± 2.56 ^{A}	13.42 ± 1.7 ^{A}	13.07 ± 0.91 ^{A}	11.06 ± 1.92 ^{A}	10.5 ± 1.45 ^{A}	9.79 ± 1.39 ^{A}
(1-methylethyl)-benzene	8	7.61 ± 0 ^{A}	1.31 ± 0.33 ^{A}	1.04 ± 0.16 ^{BC}	1.35 ± 0.37 ^{B}	1.11 ± 0.05 ^{BC}	<LOQ	<LOQ	<LOQ	<LOQ	<LOQ
2-Pinene	2	15.79 ± 3.09 ^{A}	0.9 ± 0.06 ^{B}	0.98 ± 0.28 ^{B}	0.9 ± 0.23 ^{B}	<LOQ	0.86 ± 0.13 ^{B}	<LOQ	<LOQ	<LOQ	<LOQ
1-butoxy-2-propanol	1	<LOQ	<LOQ	<LOQ	<LOQ	<LOQ	<LOQ	<LOQ	<LOQ	<LOQ	<LOQ
Dehydrosabinene	2	92.97 ± 1.22 ^{A}	<LOQ	3.65 ± 0.87 ^{B}	3.43 ± 0.83 ^{BC}	2.33 ± 0.23 ^{BCD}	2.41 ± 0.11 ^{BCD}	2.84 ± 0.43 ^{BC}	3.32 ± 0.29 ^{BC}	2.02 ± 0.22 ^{BCD}	1.93 ± 0.26 ^{CD}
2-Heptenal	6	<LOQ	0.77 ± 0.12 ^{BC}	0.86 ± 0.1 ^{BC}	0.72 ± 0.08 ^{CD}	1.12 ± 0.11 ^{A}	0.87 ± 0.07 ^{BC}	0.69 ± 0.07 ^{CD}	1.01 ± 0.12 ^{AB}	<LOQ	0.5 ± 0.05 ^{DE}
Benzaldehyde	6	4.87 ± 0.64 ^{A}	2.82 ± 0.56 ^{B}	1.4 ± 0.08 ^{C}	1.47 ± 0.05 ^{C}	1.69 ± 0.34 ^{C}	1.61 ± 0.13 ^{C}	1.11 ± 0.14 ^{C}	1.59 ± 0.16 ^{C}	1.55 ± 0.08 ^{C}	1.3 ± 0.14 ^{C}
1-Heptanol	1	0.85 ± 0.09 ^{BCD}	0.64 ± 0.1 ^{D}	0.77 ± 0.16 ^{CD}	1.43 ± 0.33 ^{AB}	1.02 ± 0.15 ^{BCD}	1.4 ± 0.26 ^{ABC}	1.86 ± 0.37 ^{A}	1.29 ± 0.24 ^{ABC}	1.19 ± 0.05 ^{BCD}	1.23 ± 0.19 ^{ABCD}
1-Octen-3-ol	3	0.57 ± 0 ^{D}	5.42 ± 0.38 ^{AB}	5.51 ± 0.66 ^{AB}	5.5 ± 0.41 ^{AB}	4.86 ± 0.69 ^{AB}	5.25 ± 0.18 ^{AB}	5.4 ± 0.1 ^{AB}	5.72 ± 0.1 ^{A}	4.63 ± 0.07 ^{B}	3.28 ± 0.18 ^{C}
2-pentylfuran	8	7.61 ± 0 ^{C}	7.05 ± 0.81 ^{C}	11.44 ± 2.27 ^{B}	16.57 ± 0.78 ^{A}	8.82 ± 0.73 ^{BC}	17.8 ± 0.26 ^{A}	17.03 ± 0.89 ^{A}	18.65 ± 1.16 ^{A}	16.68 ± 0.9 ^{A}	3.06 ± 0.43 ^{D}
Butyl butanoate	2	<LOQ	<LOQ	<LOQ	<LOQ	<LOQ	<LOQ	<LOQ	<LOQ	<LOQ	1.61 ± 0.42 ^{A}
Octanal	4	<LOQ	3.86 ± 0.11 ^{D}	3.98 ± 0.78 ^{CD}	4.94 ± 0.41 ^{ABC}	4.61 ± 0.46 ^{BCD}	5.52 ± 0.24 ^{AB}	4.19 ± 0.24 ^{CD}	5.89 ± 0.11 ^{A}	4.42 ± 0.08 ^{CD}	2.51 ± 0.36 ^{E}
3-Carene	2	7.61 ± 0 A	<LOQ	<LOQ	<LOQ	<LOQ	<LOQ	<LOQ	<LOQ	<LOQ	1.14 ± 0.32 ^{B}
p-Cymene	2	24.37 ± 3.59 ^{A}	0.92 ± 0.17 ^{B}	0.81 ± 0.07 ^{B}	<LOQ	<LOQ	<LOQ	<LOQ	0.83 ± 0.06 ^{B}	<LOQ	<LOQ
D-Limonene	5	0.21 ± 0 ^{D}	5.41 ± 0.5 ^{C}	9.06 ± 0.85 ^{AB}	9.25 ± 0.67 ^{AB}	6.14 ± 0.53 ^{C}	9.17 ± 0.52 ^{AB}	8.61 ± 1.38	10.17 ± 0.25 ^{A}	8.76 ± 0.76 ^{AB}	7.3 ± 0.35 ^{CD}
Benzenemethanol	1	1.37 ± 0.19 ^{A}	<LOQ	<LOQ	0.99 ± 0.17 ^{AB}	0.9 ± 0.16 ^{BC}	1.11 ± 0.19 ^{AB}	1.09 ± 0.12 ^{AB}	1.14 ± 0.18 ^{AB}	1 ± 0.07 ^{AB}	1.14 ± 0.12 ^{AB}
3-Octen-2-one	7	0.6 ± 0.1 ^{C}	1.03 ± 0.13 ^{A}	1.06 ± 0.12 ^{A}	1 ± 0.1 ^{A}	0.99 ± 0.16 ^{A}	1.03 ± 0.06 ^{A}	0.87 ± 0.05 ^{ABC}	1.09 ± 0.08 ^{A}	0.93 ± 0.05 ^{AB}	0.69 ± 0.09 ^{BC}
Benzeneacetaldehyde	4	<LOQ	2.14 ± 0.09 ^{CD}	2.01 ± 0.1 ^{CDE}	1.53 ± 0.22 ^{DE}	1.48 ± 0.21 ^{E}	2.46 ± 0.28 ^{C}	5.65 ± 0.13 ^{A}	1.73 ± 0.07 ^{DE}	3.98 ± 0.03 ^{B}	5.23 ± 0.56 ^{A}
2-Octenal	6	3.22 ± 0.27 ^{B}	2.97 ± 0.16 ^{BC}	2.56 ± 0.41 ^{BCD}	1.92 ± 0.21 ^{DE}	4.27 ± 0.4 ^{A}	3.02 ± 0.11 ^{BC}	1.77 ± 0.12 ^{EF}	4.07 ± 0.07 ^{A}	2.5 ± 0.11 ^{CD}	1.22 ± 0.23 ^{F}
Acetophenone	7	<LOQ	0.72 ± 0.13 ^{A}	0.59 ± 0.07 ^{ABC}	0.5 ± 0.06 ^{BC}	0.63 ± 0.11 ^{AB}	0.59 ± 0.06 ^{ABC}	0.43 ± 0.06 ^{BC}	0.5 ± 0.02 ^{BC}	0.54 ± 0.06 ^{ABC}	0.6 ± 0.08 ^{ABC}
1-Octanol	1	2.19 ± 0.28 ^{B}	2.59 ± 0.22 ^{AB}	2.59 ± 0.52 ^{AB}	2.43 ± 0.33 ^{AB}	3.34 ± 0.55 ^{A}	3.31 ± 0.41 ^{A}	2.56 ± 0.23 ^{AB}	2.6 ± 0.19 ^{AB}	2.34 ± 0.28 ^{AB}	2.69 ± 0.41 ^{AB}
Nonanal	4	737.15 ± 20.26 ^{A}	78.63 ± 9.02 ^{BC}	83.13 ± 5.71 ^{B}	52.97 ± 16.66 ^{CD}	67.4 ± 1.76 ^{BCD}	70.25 ± 3.23 ^{BCD}	55.45 ± 3.09 ^{CD}	57.63 ± 3.5 ^{BCD}	54.78 ± 3.62 ^{CD}	47.24 ± 1.56 ^{D}
Benzeneethanol	1	1.67 ± 0.34 ^{B}	<LOQ	<LOQ	<LOQ	1.28 ± 0.26 ^{B}	<LOQ	<LOQ	<LOQ	<LOQ	3.43 ± 0.32 ^{A}
2-Nonenal	6	11.14 ± 1.81 ^{A}	2.55 ± 0.21 ^{B}	4.11 ± 0.32 ^{B}	2.93 ± 0.82 ^{B}	2.64 ± 0.34 ^{B}	3.07 ± 0.09 ^{B}	3.01 ± 0.04 ^{B}	2.93 ± 0.1 ^{B}	2.87 ± 0.01 ^{B}	2.83 ± 0.11 ^{B}
2-methyl-undecane	8	<LOQ	1.24 ± 0.16 ^{B}	1.91 ± 0.45 ^{A}	1.44 ± 0.32 ^{AB}	1.7 ± 0.37 ^{AB}	1.58 ± 0.04 ^{AB}	1.75 ± 0.13 ^{AB}	1.76 ± 0.13 ^{AB}	1.39 ± 0.07 ^{AB}	1.23 ± 0.09 ^{B}
3-methyl-undecane	8	<LOQ	2.7 ± 0.42 ^{C}	3.34 ± 0.28 ^{BC}	3.67 ± 0.84 ^{ABC}	3.76 ± 0.47 ^{AB}	4.26 ± 0.1 ^{AB}	4.5 ± 0.07 ^{A}	4.48 ± 0.12 ^{A}	3.69 ± 0.04 ^{ABC}	3.88 ± 0.19 ^{AB}
p-menthan-3-ol	1	0.54 ± 0 ^{A}	<LOQ	<LOQ	<LOQ	0.54 ± 0 ^{A}	<LOQ	<LOQ	<LOQ	<LOQ	<LOQ
Naphthalene	8	<LOQ	1.13 ± 0.16 ^{C}	1.55 ± 0.25 ^{ABC}	1.52 ± 0.26 ^{ABC}	1.33 ± 0.09 ^{BC}	1.45 ± 0.1 ^{ABC}	1.63 ± 0.03 ^{AB}	1.85 ± 0.17 ^{A}	1.46 ± 0.19 ^{ABC}	1.31 ± 0.1 ^{BC}
1-Dodecene	8	<LOQ	0.9 ± 0.08 ^{CD}	1.2 ± 0.25 ^{BC}	1.29 ± 0.26 ^{BC}	1.52 ± 0.32 ^{AB}	1.35 ± 0.07 ^{ABC}	<LOQ	1.78 ± 0.12 ^{A}	1.25 ± 0.08 ^{BC}	1.32 ± 0.1 ^{ABC}
Bornyl acetate	2	39.45 ± 2.38 ^{A}	<LOQ	<LOQ	2.27 ± 0.21 ^{B}	<LOQ	<LOQ	<LOQ	<LOQ	<LOQ	<LOQ
Heptylcyclohexane	8	<LOQ	1.64 ± 0.12 ^{AB}	1.46 ± 0.26 ^{B}	1.39 ± 0.2 ^{B}	2 ± 0.33 ^{A}	1.48 ± 0.08 ^{AB}	1.48 ± 0.16 ^{AB}	1.92 ± 0.11 ^{AB}	1.45 ± 0.25 ^{B}	1.4 ± 0.11 ^{B}
Y-nonalactone	7	1.18 ± 0.24 ^{A}	<LOQ	<LOQ	<LOQ	<LOQ	<LOQ	<LOQ	<LOQ	<LOQ	<LOQ
n-Decanoic acid	2	7.61 ± 0 ^{C}	<LOQ	28.66 ± 4.45 ^{A}	<LOQ	<LOQ	<LOQ	<LOQ	<LOQ	20.54 ± 1.62 ^{B}	19.53 ± 1.84 ^{B}
cis-.alpha.-Bergamotene	5	3.06 ± 0.28 ^{A}	<LOQ	<LOQ	<LOQ	<LOQ	<LOQ	<LOQ	<LOQ	<LOQ	<LOQ
1-Pentadecene	8	7.61 ± 0 ^{A}	6.58 ± 1.08 ^{AB}	5.92 ± 0.38 ^{ABC}	5.18 ± 0.54 ^{BCD}	3.56 ± 0.53 ^{D}	<LOQ	4.86 ± 0.97 ^{BCD}	6.41 ± 0.35 ^{ABC}	4.65 ± 0.85 ^{CD}	5.23 ± 0.76 ^{BCD}
3-methyl-pentadecane	8	48.02 ± 3.36 ^{A}	3.88 ± 0.79 ^{B}	3.19 ± 0.31 ^{B}	2.73 ± 0.08 ^{B}	3.43 ± 0.25 ^{B}	3.31 ± 0.23 ^{B}	3.27 ± 0.11 ^{B}	3.61 ± 0.26 ^{B}	2.97 ± 0.15 ^{B}	3.64 ± 0.2 ^{B}

Hexadecanoic acid	2	<LOQ	<LOQ	<LOQ	<LOQ	<LOQ	<LOQ	1.64 ± 0.14 ^{A}	<LOQ	<LOQ	1.98 ± 0.5 ^{A}
Ethyl palmitate	2	<LOQ	<LOQ	<LOQ	<LOQ	<LOQ	2.27 ± 0.23 ^{D}	3.12 ± 0.61 ^{CD}	1.34 ± 0.27 ^{D}	5.2 ± 0.49 ^{BC}	12.24 ± 2.94 ^{A}
Ethyl cis,cis-9,12-octadecadienoate	2	<LOQ	<LOQ	<LOQ	<LOQ	<LOQ	1.15 ± 0.24 ^{BC}	1.42 ± 0.19 ^{BC}	<LOQ	2.15 ± 0.16 ^{B}	6.57 ± 1.36 ^{A}
(E)-9-octadecenoic acid ethyl ester	2	<LOQ	<LOQ	<LOQ	<LOQ	<LOQ	<LOQ	<LOQ	<LOQ	<LOQ	2.2 ± 0.56 ^{A}

Compounds used for quantification: 1: 2-heptanol; 2: Cumene; 3: 1-Octen-3-ol; 4: Octanal; 5: Limonene; 6: Trans-2-octenal; 7: Acetophenone; 8: Naphthalene.

Figure S1. Changes in the concentration of hexanal (A), nonanal (B), 2-hexenal (C), and 2-heptenal (D) during germination of IWG for 2, 4 and 6 days at 10, 15, and 20 ºC

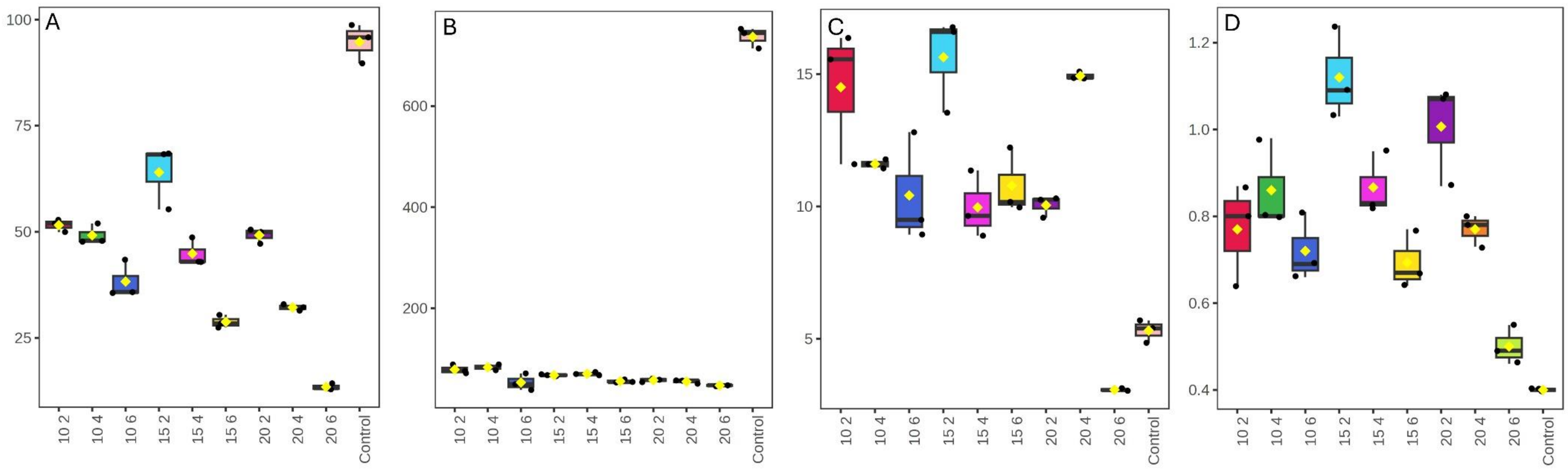

Figure S2. Variation of 1-pentanol (A), 1-hexanol (B), and 1-octenol (C) during the germination of IWG for 2, 4, and 6 days at 10 °C, 15 °C, and 20°C

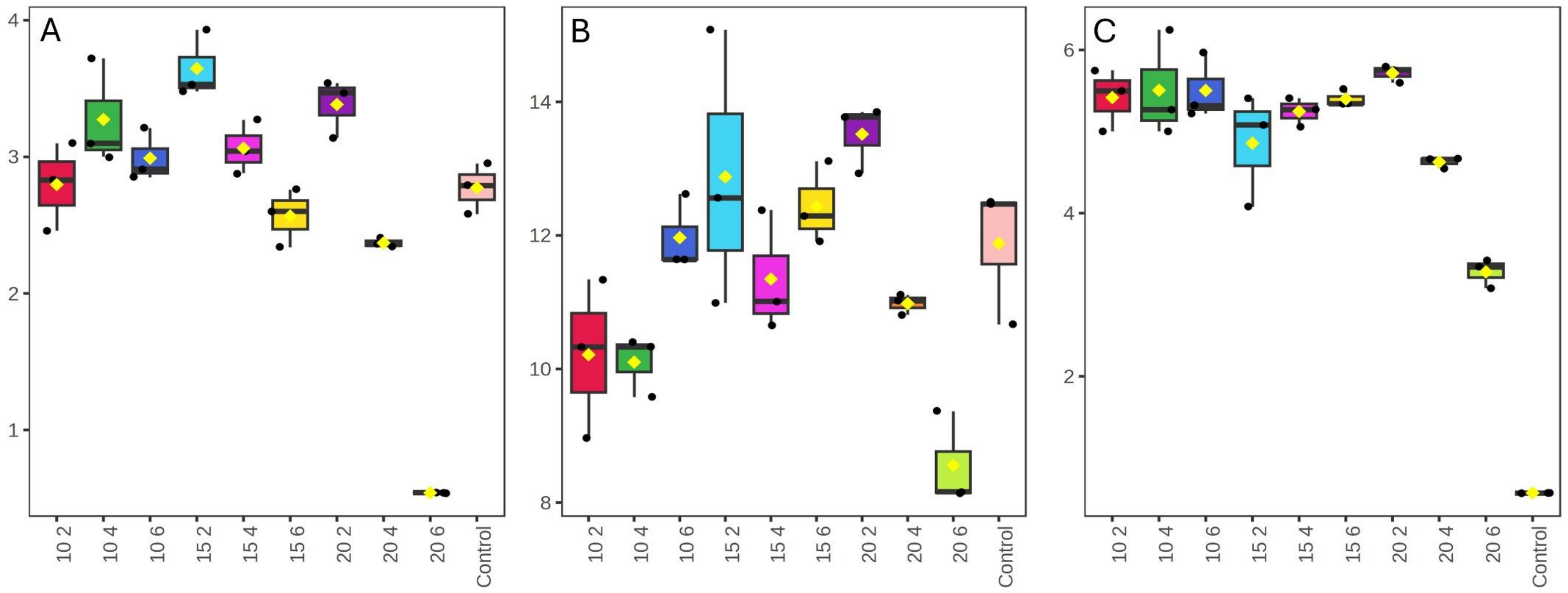

Table S3. Correlation between unsaturated fatty acids and volatiles compounds quantified in IWG samples

	Pearson correlation (r)			p-value		
Compounds	C18:3 n-3	C18:1 cis	C18:2 n-6	C18:3 n-3	C18:1 cis	C18:2 n-6
1-Pentadecene	0.02	0.18	0.20	0.926	0.342	0.300
3-methyl-butanoic	-0.19	0.20	0.27	0.304	0.285	0.156
Benzenemethanol	-0.30	-0.01	0.05	0.103	0.953	0.810
Hexanal	0.38	**0.65**	**0.60**	**0.039**	**0.000**	**0.000**
Benzaldehyde	0.18	**0.51**	**0.52**	0.339	**0.004**	**0.003**
2-Nonenal	0.30	**0.62**	**0.66**	0.105	**0.000**	**0.000**
(1-methylethyl)-be	0.18	**0.51**	**0.54**	0.330	**0.004**	**0.002**
Ethylbenzene	0.23	**0.57**	**0.61**	0.228	**0.001**	**0.000**
Styrene	0.18	**0.53**	**0.57**	0.331	**0.002**	**0.001**
Nonanal	0.19	**0.55**	**0.58**	0.306	**0.002**	**0.001**
3-Carene	0.14	**0.51**	**0.54**	0.462	**0.004**	**0.002**
2-Pinene	0.19	**0.54**	**0.58**	0.320	**0.002**	**0.001**
Y-nonalactone	0.19	**0.55**	**0.59**	0.319	**0.002**	**0.001**
Pentanoic acid	0.14	**0.51**	**0.54**	0.450	**0.004**	**0.002**
p-Cymene	0.15	**0.51**	**0.54**	0.426	**0.004**	**0.002**
3-methyl-pentadeca	0.14	**0.50**	**0.54**	0.446	**0.005**	**0.002**
Bornyl acetate	0.16	**0.51**	**0.54**	0.389	**0.004**	**0.002**
Dehydrosabinene	0.17	**0.53**	**0.56**	0.359	**0.003**	**0.001**
cis-.alpha.-Bergam	0.17	**0.53**	**0.57**	0.363	**0.002**	**0.001**
4-hydroxy-4-methyl	-0.05	-0.05	-0.11	0.785	0.779	0.577
1-butoxy-2-propano	-0.02	-0.19	-0.19	0.900	0.304	0.313
1-Hexanol	0.04	0.10	0.03	0.832	0.608	0.861
1-Pentanol	0.46	0.36	0.27	**0.010**	0.054	0.143
2-Octenal	0.13	0.40	0.27	0.477	**0.030**	0.146
3-methyl-undecane	-0.21	-0.46	-0.50	0.262	**0.010**	**0.005**
D-Limonene	-0.10	-0.44	-0.46	0.614	**0.015**	**0.010**

Naphthalene	-0.08	-0.36	-0.40	0.683	0.054	**0.031**
Heptanal	-0.11	-0.48	-0.54	0.553	**0.008**	**0.002**
Heptylcyclohexane	-0.09	-0.25	-0.34	0.648	0.182	0.069
2-methyl-undecane	0.13	-0.16	-0.24	0.487	0.409	0.201
1-Octen-3-ol	0.05	-0.36	-0.41	0.773	0.053	**0.023**
Octanal	0.00	-0.29	-0.36	0.984	0.121	0.050
1-Dodecene	0.02	0.01	-0.10	0.899	0.967	0.611
Acetophenone	0.23	0.07	0.03	0.224	0.721	0.856
1-Octanol	0.05	0.01	-0.05	0.784	0.979	0.786
2-pentylfuran	-0.01	-0.20	-0.22	0.949	0.290	0.249
2-Hexenal	0.05	-0.12	-0.19	0.800	0.523	0.309
2-Heptenal	0.20	0.06	-0.07	0.300	0.750	0.731
3-Octen-2-one	0.30	0.03	-0.05	0.113	0.873	0.779
5-methyl-2-hexanon	-0.58	-0.40	-0.39	**0.001**	**0.028**	**0.033**
Benzeneacetaldehyd	-0.44	-0.51	-0.46	**0.015**	**0.004**	**0.010**
1-Heptanol	-0.21	-0.33	-0.32	0.258	0.078	0.087
Hexadecanoic acid	-0.43	-0.43	-0.35	**0.019**	**0.019**	0.061
3-methyl-1-Butanol	-0.42	-0.41	-0.33	**0.020**	**0.025**	0.075
2,3-Butanediol	-0.43	-0.34	-0.28	**0.017**	0.066	0.128
Butyl butanoate	-0.31	-0.21	-0.17	0.099	0.274	0.366
(E)-9-octadecenoic	-0.33	-0.24	-0.19	0.072	0.194	0.308
Ethyl palmitate	-0.45	-0.33	-0.28	**0.014**	0.077	0.134
Ethyl cis,cis-9,12	-0.39	-0.28	-0.23	**0.033**	0.133	0.216
Benzeneethanol	-0.23	0.01	0.06	0.226	0.978	0.751
n-Decanoic acid	0.19	0.19	0.23	0.311	0.310	0.221

* Values in bold represent correlation higher than 0.5 and p-value lower than 0.05.
